# Antimicrobial resistance and real-time PCR detection of *blaKPC* in *Klebsiella pneumoniae* isolated from wound infections in a tertiary care hospital

**DOI:** 10.3389/frabi.2025.1700157

**Published:** 2026-01-27

**Authors:** Bader S. Alotaibi, Farkhanda Syed, Fawaz M. Almufarriji, Bilal Ahmad Tantry

**Affiliations:** 1 Department of Clinical Laboratory Sciences, College of Applied Medical Sciences, Al- Quwayiyah, Shaqra University, Riyadh, Saudi Arabia; 2 Department of Microbiology Dasmesh College, Faridkot, India; 3 Department of Microbiology, Goverment Medical College (GMC), Srinagar, India

**Keywords:** *Klebsiella pneumoniae*, antimicrobial resistance, *blaKPC* gene, real-time PCR, meropenem resistance

## Abstract

**Background:**

*Klebsiella pneumoniae* is a common Gram-negative bacterium frequently associated with wound infections. A major public health concern is the emergence of carbapenem-resistant strains, particularly those carrying the *blaKPC* gene. This study aimed to detect the *blaKPC* gene and to determine the antibiotic resistance patterns of *K. pneumoniae* isolates obtained from wound specimens in a tertiary care hospital in North India.

**Methods:**

A total of 1,080 wound swab specimens were collected between October 2023 and September 2024. The isolates were identified as *K. pneumoniae* using the VITEK-2 identification system and standard biochemical tests. Antimicrobial susceptibility was determined with the Kirby–Bauer disk diffusion method and broth microdilution, interpreted according to the Clinical and Laboratory Standards Institute (CLSI) guidelines. Carbapenemase production was assessed using the modified Hodge test. Real-time quantitative PCR (qPCR), with 16S rRNA as the internal control, was employed to detect the *blaKPC* gene in isolates resistant to meropenem.

**Results:**

Out of 560 K*. pneumoniae* isolates, 110 (19.6%) were resistant to meropenem. These resistant isolates also displayed high rates of multidrug resistance, with over 90% resistant to amikacin, ceftazidime, ampicillin, and cefazolin. The qPCR assay revealed that all 110 meropenem-resistant isolates carried the *blaKPC* gene, with PCR cycle threshold (*C*_t_) values ranging from 12 to 32. No amplification was observed in the meropenem-sensitive negative controls. The diagnostic performance of the qPCR assay demonstrated an area under the curve (AUC) of 0.99, confirming its high accuracy as a diagnostic tool. Furthermore, 83.6% of the isolates harboring the *blaKPC* gene.

**Conclusion:**

In conclusion, *K. pneumoniae* isolates exhibit a concerning rate of carbapenem resistance mediated by the *blaKPC* gene. Antimicrobial stewardship and molecular surveillance are crucial for the prevention of the spread of carbapenem-resistant *K. pneumoniae* in clinical settings.

## Introduction

Gram-negative bacteria pose a major threat to healthcare systems across the world. *Klebsiella pneumoniae* is a rod-shaped, capsulated, facultative, anaerobic Gram-negative bacterium belonging to the family Enterobacteriaceae. It can be seen in soil and water and in various hosts, including people and animals (AlTamimi et al., 2017). There is substantial mortality associated with community- or hospital-acquired infections with this bacterium, particularly in immunocompromised individuals (Gandor et al., 2022). The Centers for Disease Control and Prevention (CDC) categorized carbapenem-resistant *K. pneumoniae* (CRKP) as an urgent threat to human health in 2019, citing its resistance to such antibiotics as carbapenem (Huang et al., 2023). Carbapenem-resistant Enterobacterales (CRE) are microorganisms that destroy carbapenems or produce carbapenem (Papanikolopoulou et al., 2024).

The increasing antibiotic resistance of Enterobacterales limits the services that can be used to treat infection. Carbapenems are commonly used as a treatment measure against infections caused by organisms that are resistant to a variety of drugs (Dong et al., 2022; Darby et al., 2024). However, it is unfortunate that the bacteria have also developed defenses against this line of drugs. These are the organisms that comprise the Enterobacterales family and are resistant to carbapenem (i.e., CRE). CRE is characterized by the presence of a carbapenemase or the resistance to at least one carbapenem (van Duin and Paterson, 2020). Carbapenemase-producing Enterobacterales (CRE) are of particular concern owing to having a beta-lactamase called carbapenemase that can hydrolyze the antibiotics of the carbapenem group or resistance to a carbapenem to which the organism is not known to be naturally resistant (Tamma et al., 2024). Due to its wide range, carbapenems are typically employed only in the periodical treatment of multidrug-resistant (MDR) bacteria (Tamma et al., 2023).

The most common class A carbapenemases are *K. pneumoniae* carbapenemase (KPC), imipenem (IMI)-hydrolyzing beta-lactamase, class A non-metallo-carbapenemases (NMC-A), Guyana extended-spectrum (GES) beta-lactamase, and *Serratia marscescens* enzyme (SME) (Mutuku et al., 2022a, b). Class A carbapenemases possess a serine amino acid in the active sites and are able to cleave penicillins, cephalosporins, carbapenems, and aztreonam (AZT). The most common gene of the carbapenemase-producing CRE is *KPC* and is the most prevalent in the United States. However, they are common to people in the Middle East, Africa, Asia, South America, Spain, France, Belgium, and Romania (Albiger et al., 2015). According to the Center for Disease Dynamics, Economics, and Policy (CDDEP), up to 60% Indian *K. pneumoniae* isolates are resistant to carbapenems and 80% are resistant to cephalosporins. Parveen et al. (2010) noted that the rate of individuals resistant to carbapenem rose to 44% in 2010 in comparison to the figure in 2008, i.e., 9%.

KPC is currently an Ambler class A beta-lactamase and is the most common carbapenem-resistant enzyme occurring globally. It is also very dominant in North America and Europe. The primary origin of carbapenem resistance is the class B New Delhi metallo-lactamase (NDM) that is produced by the Ambler (Johnson and Woodford, 2013).

Clinical dissemination of the *blaKPC* gene-bearing bacteria can add to the problem of antibiotic resistance globally due to the promotion of the increase of meropenem (MEM)- and IMI-resistant strains of bacteria (Richter et al., 2012). It appears that KPC is topical due to the mistreatment and the misuse of antibiotics named as carbapenem, which is one of the risks currently posing a threat with regard to the spread of the carbapenemase enzyme. Due to an outbreak in the number of strains with the *blaKPC* gene, the KPC and OXA-48 strains can further intensify (Bina et al., 2015). The antibiotic resistance attributable to KPC is transmissible, and it spreads extremely quickly, particularly where the transferred genes are expressing carbapenemase. This causes serious pandemics and results in the number of treatments being very scarce. The most common group is the carbapenemase gene that is sharable among humans. There has been a connection between morbidity and mortality and severe KPC-KP infections treated with insufficient empirical therapy (Adhikari et al., 2018; Giacobbe et al., 2023).

There is a clinically significant global concern over carbapenem resistance in *K. pneumoniae*, in particular MEM resistance. This study was designed to assess the current distribution of the *blaKPC* gene among clinical isolates obtained from wound samples using real-time quantitative reverse transcription PCR (qRT-PCR). This approach accurately identified the carbapenemase genes in pre-validated clinical CRE isolates, demonstrating its excellent specificity and sensitivity. A simple, sensitive technique was developed for the rapid detection of clinical carbapenemases.

## Materials and methods

### Study design and sample collection

This prospective study was conducted in Dasmesh Hospital in Faridkot, India, between October 2023 and September 2024. A total of 1,080 non-duplicate wound swab specimens were collected from patients presenting with wound infections. The specimens were processed in the Microbiology Laboratory using conventional and molecular methods. The ethical principles of the World Medical Association and the Declaration of Helsinki were used in the conduct of the study to ascertain safe handling of the bacterial strains used and for the protection of the general public health. This study was approved by the Institutional Review Board of the Dasmesh Institute of Research College.

### Isolation and identification of *K. pneumoniae*

Identification of the *K. pneumoniae* isolates was done first using conventional microbiology methods according to Koshi (2001). The colony morphology, the Gram reaction, and a set of biochemical tests confirmed the large, mucoid, lactose-fermenting colony production on MacConkey agar. The final identification of the species within the bacteria was done in the automated identification system VITEK 2 (bioMerieux, Marcy-l’Étoile, France).

### Antimicrobial susceptibility testing

Antimicrobial susceptibility testing (AST) was carried out utilizing both the Kirby–Bauer disk diffusion and the broth microdilution (BMD) methods. Disc diffusion was performed on Mueller–Hinton agar, and the findings were interpreted using the Clinical and Laboratory Standards Institute (CLSI) recommendations document *M100, Performance Standards for Antimicrobial Susceptibility Testing*, *35th edition* (CLSI, 2025). The following antimicrobials were tested: ampicillin (AMP, 10 μg), AMP–sulbactam (10/10 μg), ceftazidime (CAZ, 30 μg), cefuroxime (30 μg), cefotaxime (CTX, 10 μg), cefepime (5 μg), AZT (30 μg), ertapenem (10 μg), and IMI (10 μg). The fluoroquinolones included levofloxacin (LEV, 5 μg) and ciprofloxacin (CIP, 5 μg). Gentamicin (10 μg) is an aminoglycoside. All other/cotrimoxazole (1.25/23.75 μg) disks were obtained from HiMedia (Thane, India).

### Broth microdilution

The minimum inhibitory concentrations (MICs) of carbapenem were determined using a method based on BMD. The MIC panels were produced internally in accordance with the principles set forth by the CLSI (CLSI, 2024). The MICs of CTX, CAZ, AZT, MEM, CIP, amikacin (AN), AMP, LEV, trimethoprim (TMP), nitrofurantoin (NIT), tigecycline (TIG), and tetracycline (TET) were determined. The MICs of MEM were interpreted based on the CLSI breakpoints as susceptible (≤ 4 µg/ml), intermediate (8 µg/ml), or resistant (≥ 16 µg/ml).

### Phenotypic detection of KPC production

The modified Hodge test (MHT) was used to determine whether carbapenemase production is present in the carbapenem-resistant isolates in accordance with the CLSI guidelines. The occurrence of a cloverleaf-like depression around the MEM disk was believed to denote carbapenemase activity (Zohu et al., 2018). The disks containing MEM were the negative controls. The positive control of the MHT was *K. pneumoniae* ATCC BAA-1705, while the negative control was *K. pneumoniae* ATCC BAA-1706. Spots of higher growth distortion around the MEM disks implied the formation of carbapenemase. Isolates that were determined to be KPC (*blaKPC*) producers using MIC determination and the MHT were selected for testing using real-time qPCR that targeted the *blaKPC* gene. Isolates that tested negative with these phenotypic methods were excluded from the molecular analysis.

### Quality control

Quality control was assured in the AST and the phenotypic confirmation of carbapenemase production using the MHT with standard reference strains. For the MHT, *K. pneumoniae* ATCC BAA-1705 was used as the positive control strain and *K. pneumoniae* ATCC BAA-1706 as the negative control strain, while MEM disks were used as the test antibiotic.

### Genomic DNA extraction

Extraction of plasmid DNA was according to the manufacturer, performed using the QIAprep Spin Miniprep Kit (QIAGEN, Hilden, Germany). Briefly, 2 ml of an overnight bacterial culture was centrifuged at 10,000 × *g* for 5 min and a cell pellet obtained. The supernatant was disposed of and the pellet suspended in 250 µl of buffer P1 in the presence of RNase A to remove the contamination of RNA. This was followed by lysing the content with 250 µl of buffer P2 and subsequent incubation for 5 min. Centrifugation was performed, followed by 350 μl of buffer N3, which neutralizes the lysis. Following the loading of a clear supernatant on a QIAprep Spin Column, the column was then successively washed with 500 μl of buffer PB and 750 μl of buffer PE. Any excess buffer was removed by centrifugation. Of buffer EB (10 mM Tris–Cl, pH 8.5), 60 μl was used to elute the plasmid DNA. Its concentration and purity were assessed using a NanoDrop spectrophotometer (Thermo Fisher Scientific, Waltham, MA, USA), and the samples were stored at −20°C until required.

### Primer and probe sequences

The TaqMan Gene Expression Master Mix (lot no. 4369016; Thermo Fisher Scientific, Waltham, MA, USA), which contains AmpliTaq Gold DNA Polymerase Ultra Pure, was used for real-time PCR with primers and probes targeting the *blaKPC* gene: forward primer 5′-GGC CGC CGT GCA ATA C-3′, reverse primer 5′-GCC GCC CAA CTC CTT CA-3′, and FAM probe 5′-TG ATA ACG CCG CCG CCA ATT TGT-3′. 16S rRNA served as the internal control using the forward primer 5′-TGG AGC AGC ATG TGG TTT AAT TCG A-3′, the reverse primer 5′-TGC GGG ACT TAA CCC AAC AAC AAC A-3′, and the CY5 probe 5′-CA CGA GCT GAC GAC GAC ARC CAT GCA-3′.

### Detection of the *blaKPC* gene by real-time PCR

All reagents were prepared and treated on ice to preserve the activity of the enzymes and to prevent degradation. The primer and probe mixture was prepared by combining 20 µM of each primer and 10 µM of each probe. The master mix was made on ice with 10 µl of the Probe PCR Master Mix, 5 µl of the primer–probe mix, and 3 µl of sterile water prepared on ice for every 20 µl reaction. Once the master mix was thoroughly mixed, 18 µl of this mixture was added into a 96-well PCR plate. A 2-µl DNA template was added to each of the reaction wells.

### Thermal cycle conditions

Real-time PCR was carried out according to the CDC protocol, with an initial denaturation step at 95°C for 3 min, denaturation at 95°C for 3 s, and annealing/extension at 60°C for 30 s (40 cycles). Amplification was regarded positive only if the cycle threshold (*C*_t_) value was between 10 and 32. The amplification of the 16S rRNA gene detected between cycles 31 and 40 and the no-template control (NTC) had to exhibit no amplification of the target genes (*C*_t_ > 40).

### Sensitivity and specificity

The analytical sensitivity of the assay was also determined with real-time PCR at 10-fold serial dilutions of the *K. pneumoniae* cultures harboring the KPC, starting at an initial concentration of 8.0 × 10^7^ CFU/ml. The sensitivity of the assay was high in terms of analysis as reduction in the bacterial loadings allowed the target gene to be detected. The phenotypic reference tool was MHT, also described as MHT. The MHT was applied to compare the sensitivity and the specificity of the real-time PCR assay. The real-time PCR test, as a method for the detection of *blaKPC* in inculcated isolates, proved to be fast and extremely specific, with a turnaround time of 2–3 h.

## Results

### Distribution and identification of *K. pneumoniae* from wound infections

**Figure 1:**
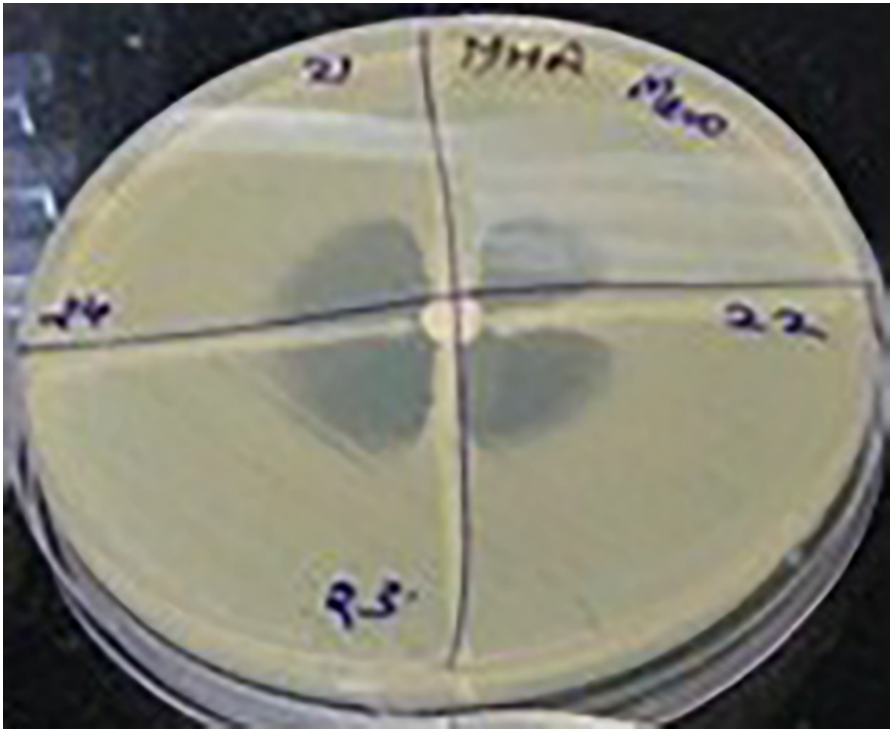
Modified Hodge test showing the phenotypic characterization of the carbapenemase-producing *Klebsiella pneumoniae* clinical isolates.

Of the total 1,080, only 560 (51.8%) *K. pneumoniae* samples were examined. Of these, 19.6% (*n* = 110) of the isolates were found to be KPC producers. The initial identification also involved Gram staining, routine biochemical testing, and colony examination. The determination of the first antimicrobial susceptibility of all the isolates of *K. pneumoniae* was performed with the Kirby–Bauer disk diffusion procedure using IMI and MEM. reduced sensitivity to carbapenems (with minimum zone diameter less than that of the CLSI 2023 interpretation of breakpoints) to carbapenems were additionally assessed using BMD. Resistant isolates were then characterized phenotypically with regard to carbapenemase activity. The MHT was used to validate the carbapenemase production phenotypically (**Figure 1**, MHT). These isolates were then chosen for the *blaKPC* gene-focused molecular investigation.

### Antimicrobial susceptibility pattern of *K. pneumoniae*

In this study, a total of 560 K*. pneumoniae* clinical isolates were used to examine their susceptibility to antimicrobial agents. Their resistance, on an individual antimicrobial agent basis, is shown in **Table 1**. The AMP resistance rates were found to be the highest (9905%). This was followed by AZT (85.0%), CAZ (85.0%), cefuroxime (80.0%), LEV (77.0%), and ceftriaxone (74.0%). The resistance levels of CIP (63.0%) and TMP–sulfamethoxazole (60.0%) were also high. There were higher susceptibility rates with the carbapenems IMI (44.0%) and MEM (44.0%), as well as gentamicin (54.0%) and AN (60.0%).

**Table 1:** MIC values of 110 ioslates.

S. no.	Clinical Isolates	MEM	CAZ	CTX	AZT	CIP	AN	AMP	TMP	TIG	TET	NIT	LEV
1	KPC001	32	>32	>32	>32	>32	>32	>32	>32	>8	>16	>16	32
2	KPC002	>32	>32	>32	>32	>32	>32	>32	>32	2	0.5	4	2
3	KPC003	4	0.25	4	0.25	0.06	1	8	0.25	2	0.5	4	2
4	KPC004	>32	>32	>32	>32	>32	>32	>32	>32	4	1	16	16
5	KPC005	>32	>32	>32	>32	>32	>32	>32	>32	>8	>16	>16	16
6	KPC006	>16	16	16	4	>16	>16	>32	>16	2	2	8	4
7	KPC007	>16	>16	>16	>16	>16	>16	>32	>16	2	0.125	16	4
8	KPC008	8	>16	16	>16	>16	>16	>32	>16	1	0.25	16	2
9	KPC009	16	>16	>16	>16	>16	>16	>32	>16	2	0.5	4	0.25
10	KPC010	4	16	16	0.5	>16	>16	>32	>16	0.5	0.125	4	2
11	KPC011	8	0.125	0.03	>16	0.03	0.5	16	0.06	0.5	0.125	8	8
12	KPC012	>16	>16	>16	>16	>16	>16	>32	>16	1	0.5	8	16
13	KPC013	8	>16	16	16	>16	>16	>32	16	1	8	16	32
14	KPC014	4	>16	4	>16	>16	>16	>32	>16	2	8	16	32
15	KPC015	4	>16	>16	16	>16	>16	>32	>16	0.3	4	16	0.5
16	KPC016	>16	>16	>16	>16	>16	>16	>32	>16	>8	>16	>16	16
17	KPC017	>16	16	16	8	>16	>16	>32	>16	1	0.25	4	0.13
18	KPC018	>16	>16	>16	>16	>16	>16	>32	>16	0.5	2	8	2
19	KPC019	>16	>16	>16	>16	>16	>16	>32	>16	0.5	0.125	2	2
20	KPC020	8	0.06	0.03	4	0.125	0.25	8	0.03	1	2	4	>16
21	KPC021	8	>16	>16	4	>16	>16	>32	0.03	1	8	>16	>16
22	KPC022	>16	>16	>16	>16	>16	>16	>32	>16	0.5	0.5	2	0.05
23	KPC023	4	0.06	8	4	1	4	16	1	4	>16	>16	8
24	KPC024	8	8	8	4	>16	2	16	>16	0.3	1	4	8
25	KPC025	>16	>16	>16	>16	>16	>16	>32	>16	2	2	8	16
26	KPC026	4	16	16	8	>16	>16	>32	>16	2	8	16	8
27	KPC027	>16	>16	>16	>16	>16	>16	>32	>16	8	>16	>16	>32
28	KPC028	16	>32	>32	>32	>32	>32	>32	>32	0.5	2	4	0.5
29	KPC029	32	>32	>32	>32	>32	>32	>32	1	2	4	16	16
30	KPC030	>32	>32	>32	>32	>32	>32	>32	>32	4	16	>16	>16
31	KPC031	>32	>32	>32	>32	>32	>32	>32	>32	1	4	16	>16
32	KPC032	>32	>32	>32	>32	>32	>32	>32	1	1	4	>16	>16
33	KPC033	>32	>32	>32	>32	>32	>32	>32	>32	2	1	4	2
34	KPC034	>32	>32	>32	>32	>32	>32	>32	>32	8	>16	>16	8
35	KPC035	16	16	>32	>32	>32	>32	>32	>32	>8	>16	>16	8
36	KPC036	>32	>32	>32	>32	>32	>32	>32	>32	>8	>16	>16	>32
37	KPC037	>32	>32	>32	>32	>32	>32	>32	>32	>8	>16	>16	32
38	KPC038	16	16	16	16	>32	8	>32	>32	1	0.5	4	2
39	KPC039	32	>32	>32	>32	>32	>32	>32	>32	2	2	8	8
40	KPC040	4	>32	>32	>32	>32	>32	>32	>32	>8	>16	>16	8
41	KPC041	>32	>32	>32	>32	>32	>32	>32	>32	>8	>16	>16	8
42	KPC042	>32	>32	>32	>32	>32	>32	>32	>32	>8	>16	>16	8
43	KPC043	4	>32	>32	>32	>32	>32	>32	>32	>8	>16	>16	8
44	KPC044	>16	32	32	32	32	32	>32	32	>8	>16	16	16
45	KPC045	>16	>16	>32	>32	32	>32	>32	>16	2	>16	>16	32
46	KPC046	>16	>16	>32	>32	32	>32	>32	>16	>4	>16	>16	>32
47	KPC047	>16	>16	>32	>32	32	>16	>32	16	2	>16	8	16
48	KPC048	4	0.125	32	0.25	0.5	>16	8	4	2	32	32	16
49	KPC049	>16	>16	32	>32	>32	>16	>32	>16	>4	8	16	16
50	KPC050	>16	>16	>32	32	>32	>16	>32	>16	4	1	>16	16
51	KPC051	8	<0.03	0.06	>16	0.06	>16	8	2	4	1	>16	8
52	KPC052	>16	>16	>32	>16	>32	>32	>32	>16	1	>16	>16	8
53	KPC053	>16	>16	>32	>16	>32	>32	>32	>16	>4	>16	>16	16
54	KPC054	>16	0.25	0.5	0.5	0.125	>16	4	4	4	>16	>16	32
55	KPC055	>16	>32	32	>16	>16	>16	>64	>32	>8	>32	>32	32
56	KPC056	4	>32	>32	>16	>16	>16	>64	>32	0.5	>32	16	32
57	KPC057	32	>32	>32	16	>16	>16	>64	>16	>4	16	>32	32
58	KPC058	>32	>32	>32	>16	>16	>16	64	>16	>8	>32	>32	>32
59	KPC059	>32	>32	>32	16	0.03	0.06	8	>16	>8	16	>32	<4
60	KPC060	16	>32	>32	>16	>16	>32	>64	>16	>8	>32	16	<4
61	KPC061	>32	>32	>32	8	16	>32	64	>16	2	>32	>32	<4
62	KPC062	>32	>32	>32	>16	>16	>16	>64	>16	2	16	>32	<4
63	KPC063	>32	>32	>32	>16	>16	>16	>64	>16	>4	>32	>32	32
64	KPC064	>32	>32	32	4	16	>16	>64	>16	>4	>32	>32	>32
65	KPC065	<2	16	32	4	16	>32	8	4	4	<2	>32	<2
66	KPC066	>32	>32	32	>16	>16	>32	>64	>16	4	4	<2	32
67	KPC067	<2	>32	16	4	>16	>16	>64	>16	2	<2	>32	16
68	KPC068	>32	>32	32	4	16	>16	>64	>16	>8	8	>32	32
69	KPC069	>32	>32	32	>16	>16	>16	>64	>32	>4	>32	>32	32
70	KPC070	<2	<2	2	8	16	>16	>64	>32	4	<2	>32	>32
71	KPC071	>64	>64	>32	>16	16	>16	>64	>16	>8	>64	>64	>64
72	KPC072	>32	>32	>32	>32	>16	>32	>64	>16	>4	>32	>32	>32
73	KPC073	16	>32	>32	>32	>16	>32	>64	>16	2	>32	>32	>32
74	KPC074	>32	>32	>32	>32	>16	>16	>64	>16	2	>32	>32	>32
75	KPC075	8	>32	>32	>32	>16	>16	>64	>16	>4	>32	>32	>32
76	KPC076	>32	16	32	>32	>32	>32	>64	>16	2	4	>32	32
77	KPC077	>32	4	2	>32	>32	>32	32	>32	>8	>16	>32	32
78	KPC078	4	>32	16	>32	>32	>16	32	>32	>8	>32	>32	32
79	KPC079	8	>32	32	>32	>32	>32	32	>32	>8	>32	>32	32
80	KPC080	8	>32	>32	>32	>32	>32	>64	>32	>8	32	>32	16
81	KPC081	>64	>64	>32	>32	>32	>32	64	>32	>8	>64	>64	>64
82	KPC082	8	<2	2	16	>32	>32	16	>32	2	<2	>32	<2
83	KPC083	4	>32	32	>32	>32	>32	32	>32	>8	>32	32	16
84	KPC084	>32	>32	>32	>32	>32	>32	>64	>16	>8	>32	>32	>32
85	KPC085	16	>32	>32	>32	>32	>16	>64	>16	>4	32	32	16
86	KPC086	8	>32	>32	>32	>32	>16	>64	>32	2	>32	>32	>32
87	KPC087	16	16	>32	>32	>32	>32	>64	>32	>8	16	>32	16
88	KPC088	8	>32	>32	32	>32	>32	>64	>32	2	>32	>32	16
89	KPC089	>32	>32	>32	32	>32	>16	>64	>32	2	2	>32	>32
90	KPC090	16	>32	>32	32	>32	>32	>64	>32	1	2	>32	4
91	KPC091	32	>32	>32	16	>32	>32	>64	>16	4	>32	>32	>32
92	KPC092	>32	>32	>32	32	32	>16	>64	>16	>4	>32	>32	32
93	KPC093	32	>32	>32	32	16	>16	32	>32	8	2	>32	0.5
94	KPC094	8	>32	>32	32	32	>32	32	>32	>4	>32	>32	>32
95	KPC095	8	0.5	0.25	32	0.125	>32	16	>32	2	0.25	4	0.5
96	KPC096	32	32	32	>32	32	>16	32	>16	>4	>16	>32	>32
97	KPC097	>16	>8	32	>32	16	>16	>64	>16	4	>8	>16	2
98	KPC098	>16	>8	16	32	16	>16	>64	>32	4	>8	>16	4
99	KPC099	>16	>8	>32	>32	32	>16	>64	>32	4	>8	>16	4
100	KPC100	>16	>8	>32	>32	>32	>16	>64	>32	4	>8	>16	4
101	KPC101	>16	>8	>32	>32	>32	32	>64	>16	2	>8	>16	4
102	KPC102	>16	>8	16	32	>32	>32	>64	>16	2	>8	>16	4
103	KPC103	>16	>8	>32	32	>32	>32	>64	>32	2	>8	>16	2
104	KPC104	>16	>8	16	32	>32	>32	>64	>32	2	>8	>16	4
105	KPC105	>16	>8	32	16	>32	>32	>64	>32	4	>8	>16	4
106	KPC106	>16	>8	32	16	>32	>32	>64	>16	4	>8	>16	2
107	KPC107	>32	>32	>32	>32	>32	>32	64	>16	4	0.25	16	>32
108	KPC108	>16	>32	>32	>32	0.125	0.5	8	2	4	0.5	>32	16
109	KPC109	8	>32	>32	32	16	32	32	>16	>32	0.063	>32	16
110	KPC110	>32	>32	16	>32	32	32	32	>16	>32	0.5	>32	>32

**Figure 2:**
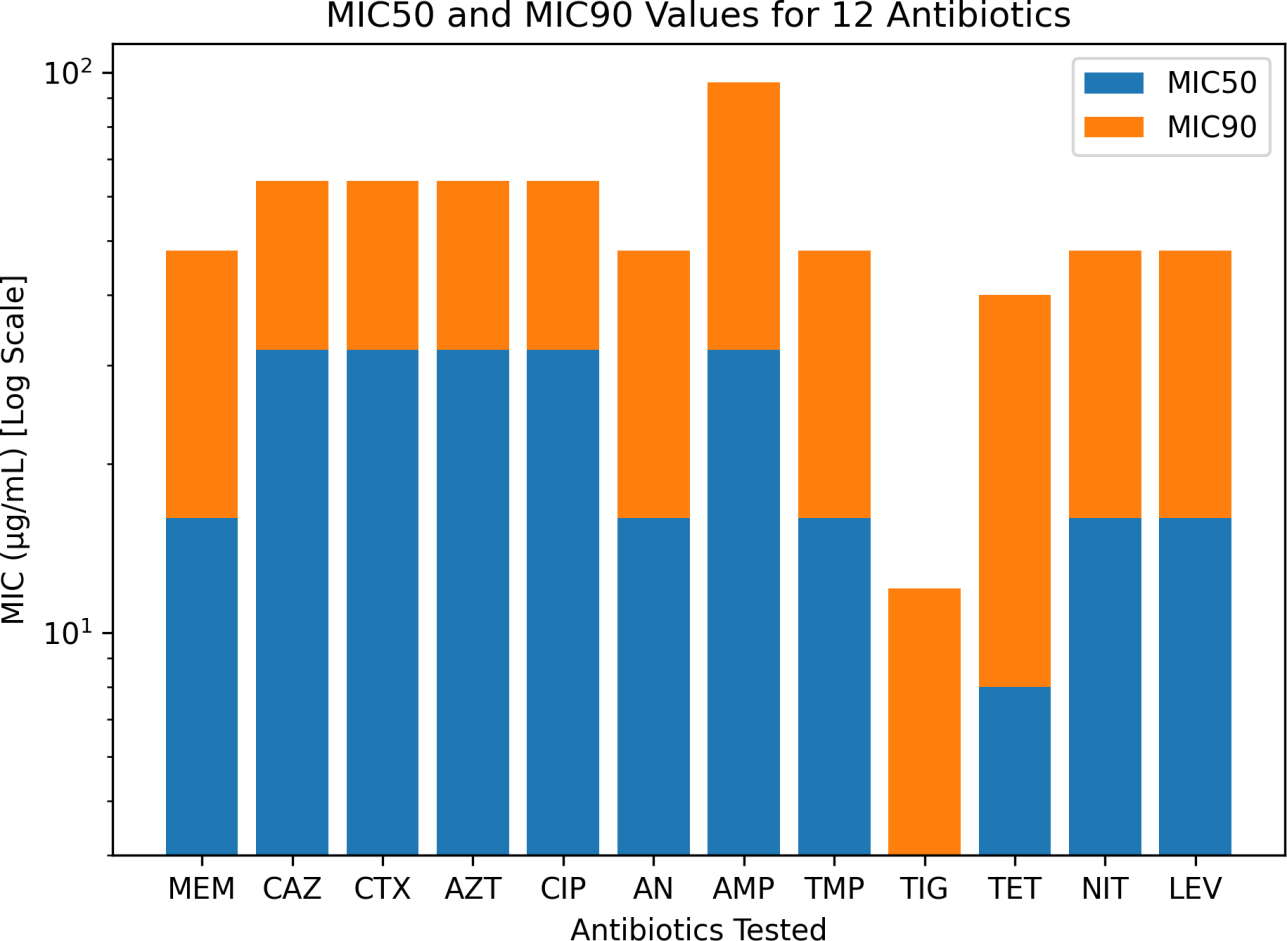
Antibiotic resistance patterns of the *Klebsiella pneumoniae* clinical isolates (*n* = 110). The percentage of resistant isolates is shown for each antibiotic tested, illustrating the resistance landscape across multiple antimicrobial classes.

**Figure 3:**
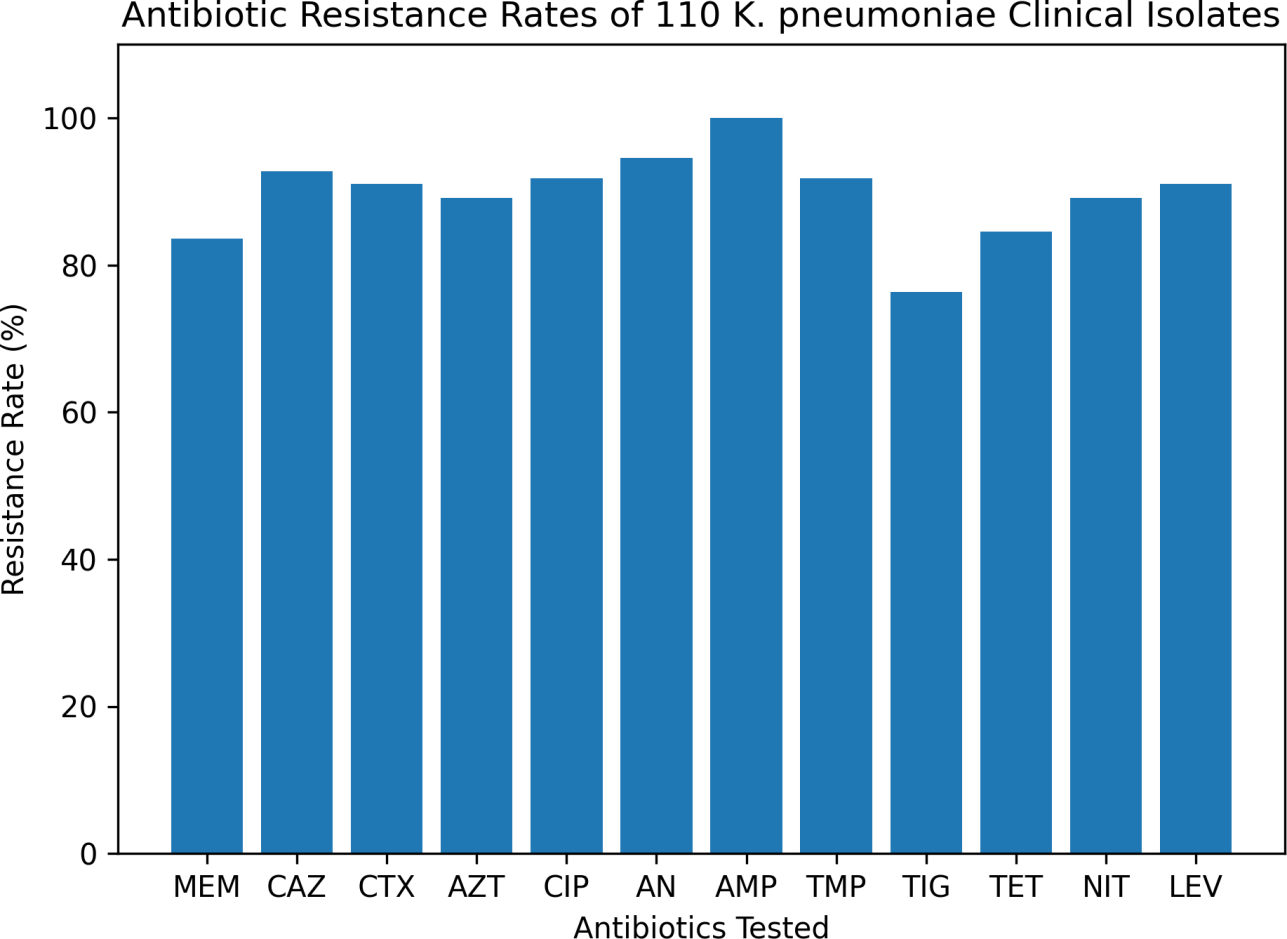
Minimum inhibitory concentration (MIC) interpretation for the 110 *Klebsiella pneumoniae* clinical isolates against 12 antibiotics: meropenem (*MEM*), ceftazidime (*CAZ*), cefotaxime (*CTX*), aztreonam (*AZT*), ciprofloxacin (*CIP*), amikacin (*AN*), ampicillin (*AMP*), trimethoprim (*TMP*), tigecycline (*TIG*), tetracycline (*TET*), nitrofurantoin (*NIT*), and levofloxacin (*LEV*).

**Figure 4:**
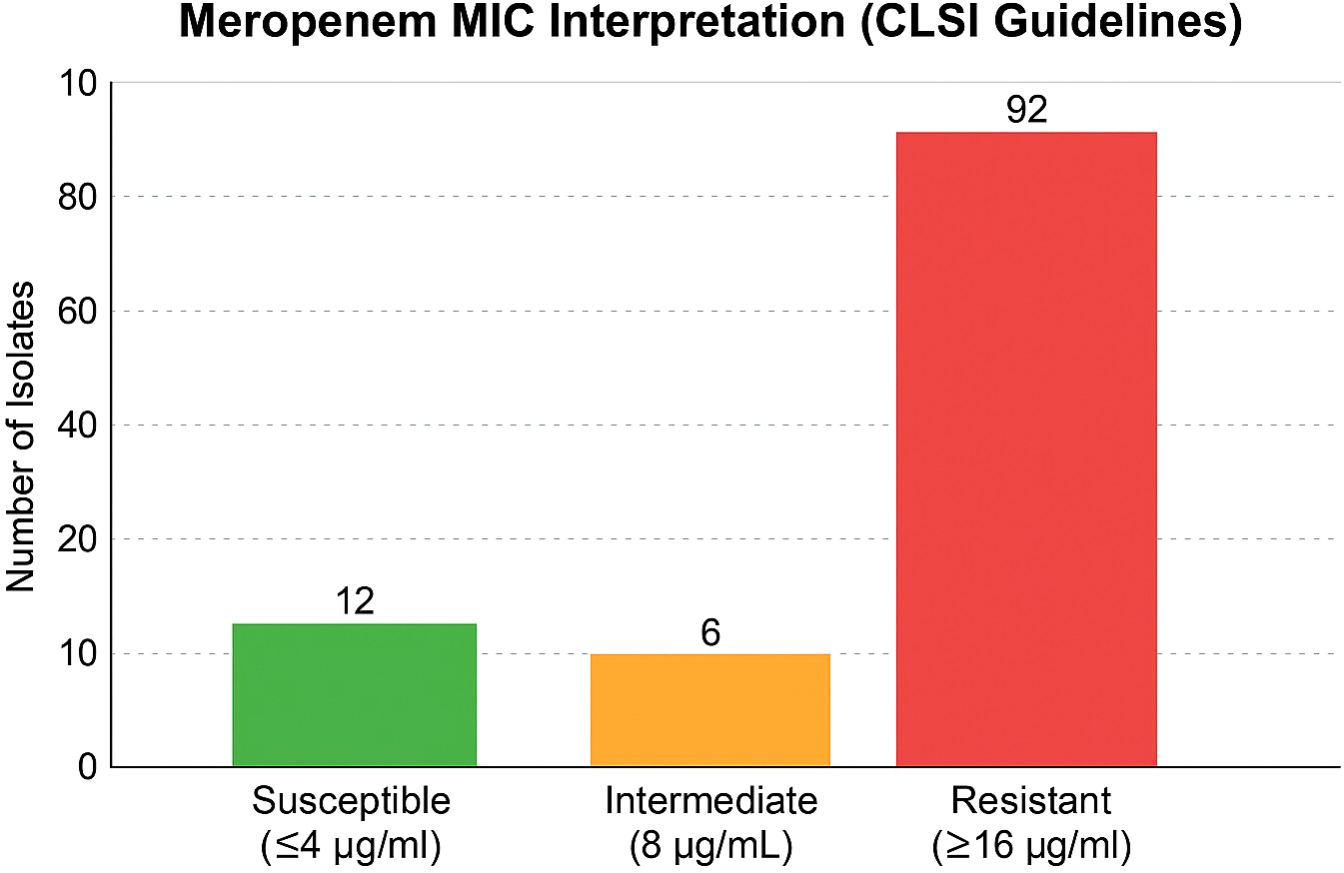
Minimum inhibitory concentration (MIC) interpretation of the *Klebsiella pneumoniae* isolates specifically against meropenem (MEM), demonstrating the distribution of the susceptible, intermediate, and resistant categories.

**Figure 5:**
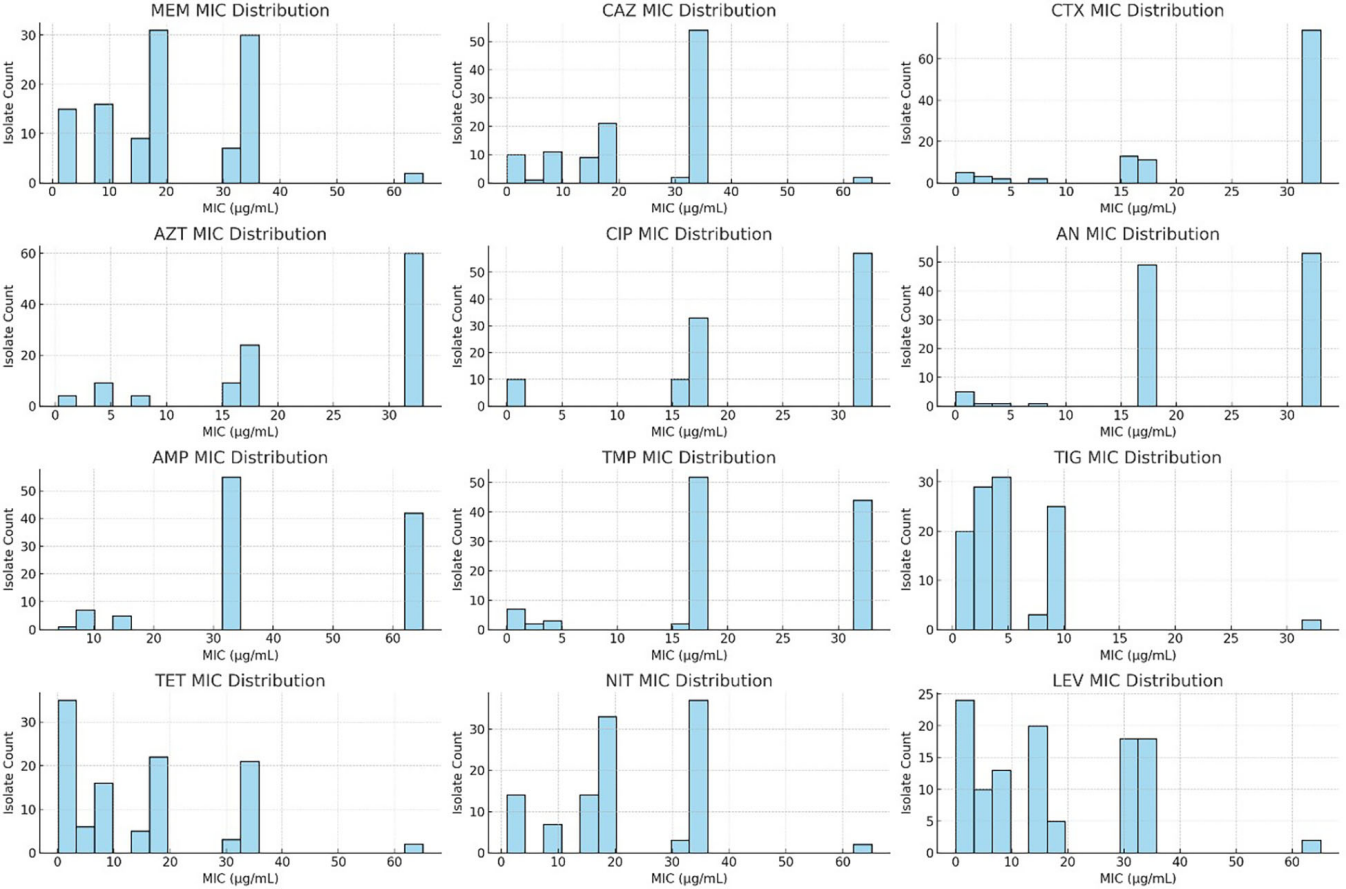
Minimum inhibitory concentration (MIC) distribution of the *Klebsiella pneumoniae* clinical isolates against 12 commonly used antibiotics. The histogram plots display the MIC values (in micrograms per milliliter) for the 110 isolates tested against meropenem (MEM), ceftazidime (CAZ), cefotaxime (CTX), aztreonam (AZT), ciprofloxacin (CIP), amikacin (AN), ampicillin (AMP), trimethoprim (TMP), tigecycline (TIG), tetracycline (TET), nitrofurantoin (NIT), and levofloxacin (LEV). Values reported as “>*X*” were transformed (e.g., “>32” as 33) to allow histogram visualization. Elevated MICs, particularly for β-lactams and fluoroquinolones, indicate multidrug resistance trends.

Among the 110 clinical isolates of *K. pneumoniae*, high resistance was observed against AMP (100%), AN (94.5%), CAZ (92.7%), CIP (91.8%), TMP (91.8%), CTX (91.0%), and AZT (89.1%). Significant resistance was also noted for LEV (91.0%), NIT (89.1%), TET (84.5%), and TIG (76.3%). The resistance rates were comparatively lower for some other antibiotics (**Figure 2**). The MIC interpretation against the 110 clinical *K. pneumoniae* isolates is shown in **Figure 3**. In addition, **Figure 4** shows the distribution of the MICs of MEM alone, out of which 12 (10.9%) isolates had low MICs (≤4 μg/ml), 6 (5.4%) isolates had intermediate resistance (MIC = 8–16 μg/ml), and 92 (83.6) isolates displayed high resistance (MICs > 16 μg/ml). Histogram plots were used to represent the distribution of the MICs (in micrograms per milliliter) for the 110 K*. pneumoniae* clinical isolates tested against 12 antibiotics (**Figure 5**). The MICs from the BMD are presented in a **Supplementary Table**.

### Meropenem susceptibility and KPC production in *K. pneumoniae*

Of the 560 clinical isolates of *K. pneumoniae* evaluated, 110 (19.6%) isolates were resistant to MEM. Real-time PCR confirmed the presence of the *blaKPC* gene. On the other hand, 450 (80.4%) isolates were susceptible to the antibiotic, and none of them possessed the *blaKPC* gene.

### Real-time PCR detection of the *blaKPC* gene

**Figure 6:**
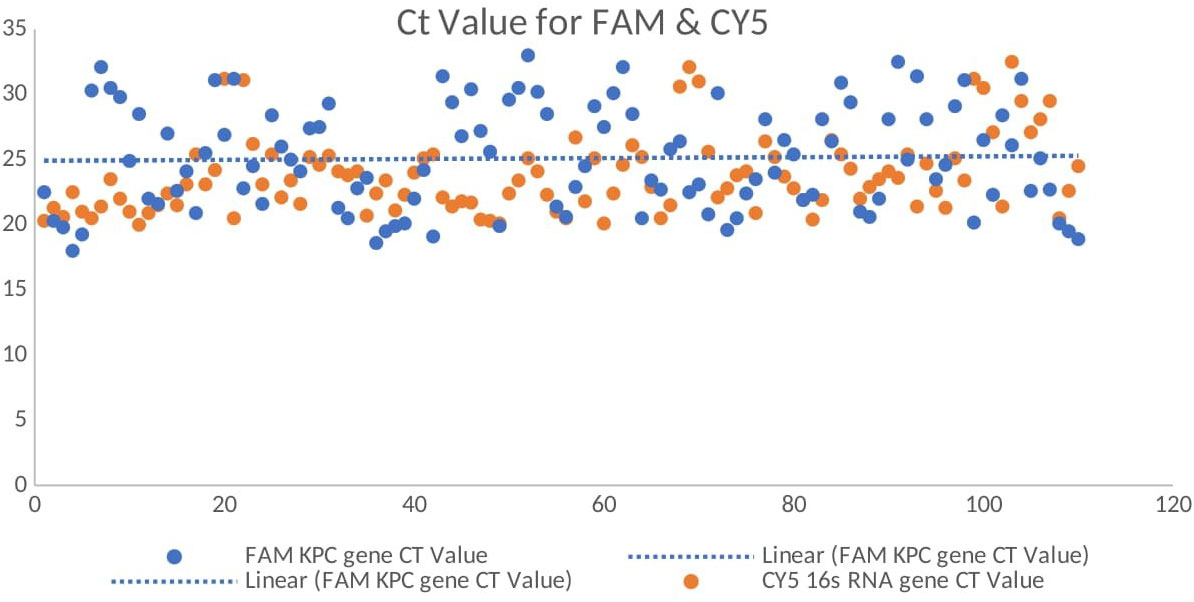
Real-time quantitative reverse transcription PCR (qRT-PCR) amplification curves showing the *C*_t_ values for the *blaKPC* gene (FAM channel) and the 16S rRNA gene (CY5 channel, internal control) among the *Klebsiella pneumoniae* isolates.

**Figure 7:**
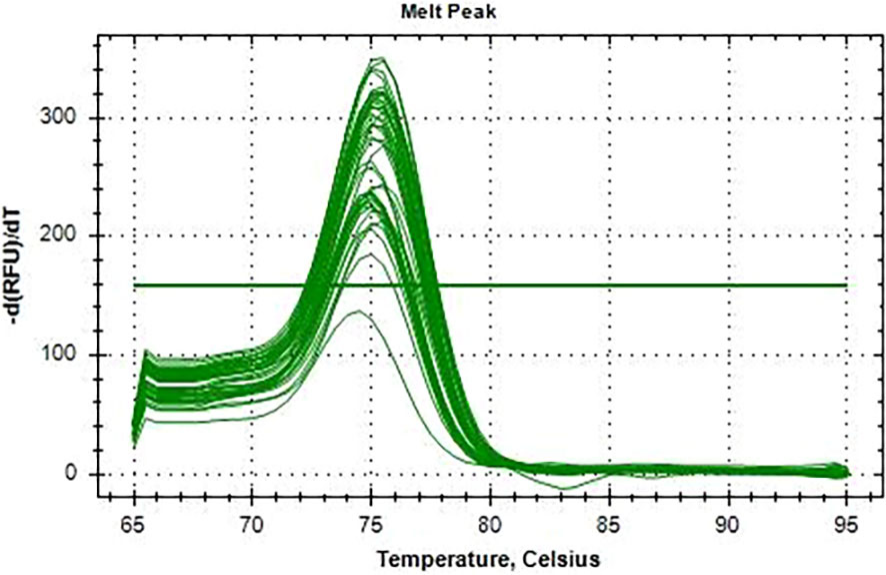
Melting curve analysis of the *blaKPC* amplicons from the 110 *Klebsiella pneumoniae* isolates. A single melt peak at ~77°C confirms the specificity of the amplified product.

All 110 K*. pneumoniae* isolates were successfully amplified utilizing primers and probes that targeted the *blaKPC* gene using real-time qPCR on the CFX 96 System (Bio-Rad, Hercules, CA, USA). Positive amplification of the *blaKPC* gene was observed, with *C*_t_ values ranging from 12 to 32 confirming the molecular presence of the resistance gene (**Figure 6**). No amplification was observed in the NTCs, confirming the absence of contamination. Amplification of 16S rRNA consistently occurred in all template wells. A FAM-labeled probe was used to detect the *blaKPC* gene, while a CY5-labeled probe was used to detect the 16S rRNA gene, which served as an internal control. A distinct melt peak was observed at approximately 77°C, confirming the specificity of the amplified product. The uniform melting profiles across samples further validated the consistency and specificity of amplification. No nonspecific products or primer–dimers were detected. The melt curves were generated using the CFX96 Touch™ Real-Time PCR Detection System (Bio-Rad, Hercules, CA, USA) (**Figure 7**). The *C*_t_ values for the 110 isolates are shown in a **Supplementary File**.

**Figure 8:**
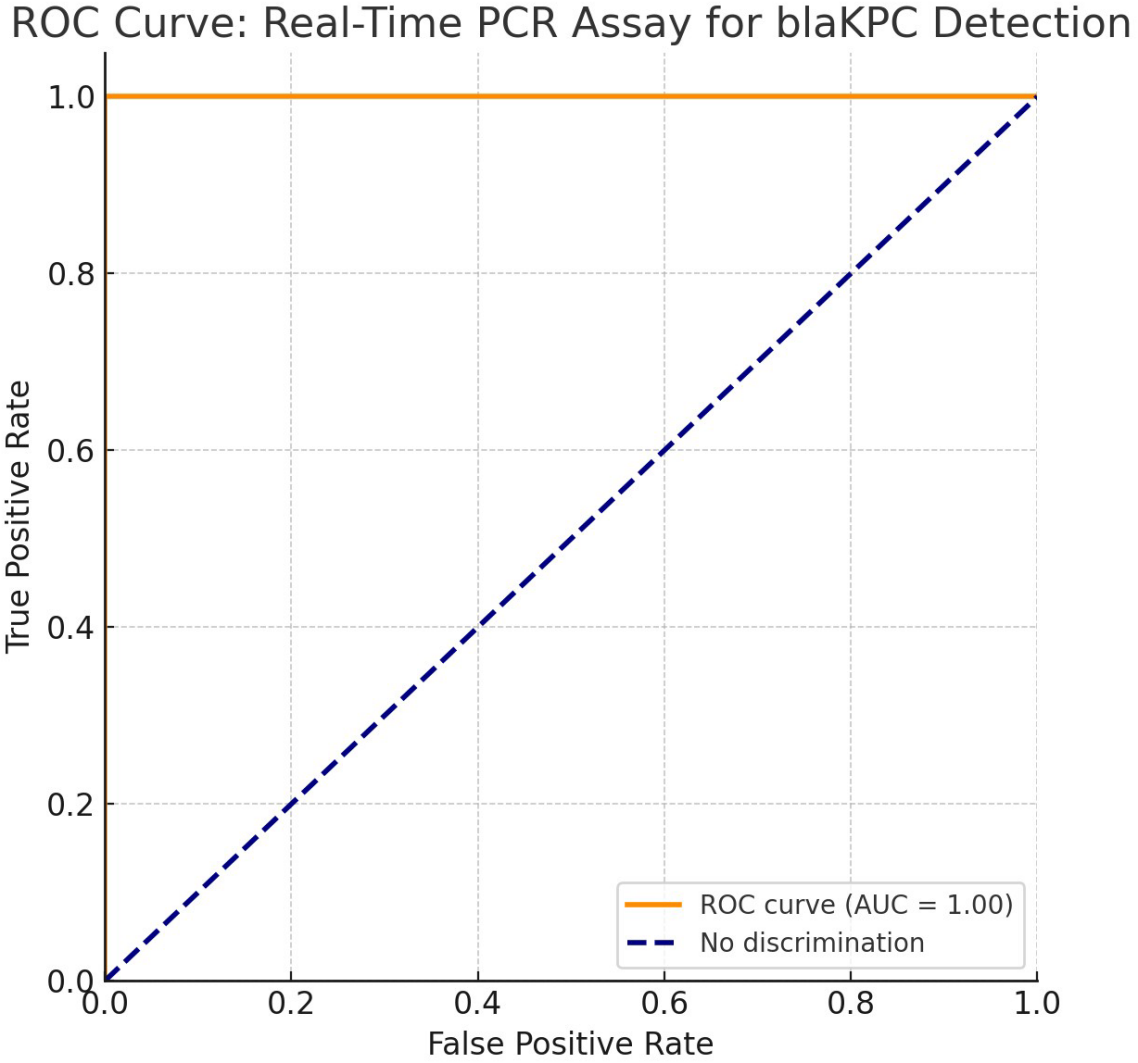
Receiver operating characteristic (ROC) curve demonstrating the excellent diagnostic performance of the quantitative PCR (qPCR) assay, with an area under the curve (AUC) of 0.99, indicating near-perfect ability to distinguish between *blaKPC*-positive and *blaKPC*-negative isolates.

The receiver operating characteristic (ROC) curve exhibited exceptional diagnostic performance, with an area under the curve (AUC) of 0.99 and nearly perfect sensitivity and specificity. It was possible to distinguish between isolates that were *blaKPC*-positive and *blaKPC*-negative using real-time PCR assay. **Figure 8** displays the sensitivity analysis results.

## Discussion

A serious health concern that affects disease control in both industrialized and developing nations due to the high rates of antibiotic abuse and improper infection control is the increase in CRKP infections. In this study, the proportion of *K. pneumoniae* isolates that expressed *blaKPC* was 19.6%. KPC is the most widespread carbapenemase reported in India. Veeraraghavan et al. (2017) found an increase in *K. pneumoniae* carbapenem resistance from 9% to 14% in India during 2008 and 2010. Albiger et al. (2015), as well as the CDDEP, reported more than 60% resistance in Indian isolates and highlighted the widespread presence of *blaKPC* and *blaNDM* as two of the most common carbapenemase genes. It is important that hospitals in India monitor the prevalent carbapenemases in every region in order to demonstrate shifts in the trend from NDM enzymes to OXA-48. Greece has the highest rate of carbapenem resistance in the world, at 68%. This is followed by India and the Eastern Mediterranean, with resistance rates of 54% each (WHO 2014). The carbapenemase mechanisms of carbapenem resistance and KPC have particular limited options in the management of infections due to the reduced treatment options with antimicrobials (Lan et al., 2021).

The results in this study showed a high rate of resistance to the major antimicrobials: AMP (100%), CAZ (92.7%), CIP (91.8%), and AZT (89.1%). These results correlate with other studies that found high multidrug resistance in KPC-producing Enterobacterales (Mutuku et al., 2022; Tamma et al., 2023; Bashir et al., 2023). Notably, our findings showed significantly reduced resistance rates to aminoglycosides (gentamicin, 54%; AN, 60%) and carbapenems (IMI, 44%; MEM, 44%) compared with those of other regional studies (Albiger et al., 2015), implying potential institutional discrepancies with regard to the antimicrobial prescribing habits and the infection control procedures.

We demonstrated the potential utility of a real-time PCR assay that specifically detects the *blaKP* gene, which resolved these challenges and showed an unmatched accuracy, with a *C*_t_ value range of 12–32 and an AUC of 0.99. This supports the results of Wang et al. (2012), who showed that diagnostic tests based on molecular techniques yield better results than traditional phenotypic tests, especially with regard to the early identification and control of KPC-producing strains in hospital environments.

The precision of the molecular recognition of the *blaKPC* pathogen using qPCR is swift, with a turnaround time of 2–3 h, which is critical for the adoption of a suitable treatment. We are in agreement with the recent studies by Mangold et al. (2011) and Hindiyeh et al. (2008) on the importance of molecular technologies in early-stage diagnoses in the context of preventing the progression of infectious diseases. Furthermore, the fact that the 16S rRNA gene was amplified in each reaction indicates that there is no PCR inhibition and that the bacterial DNA is still intact. The rapid detection of *blaKPC* genes is of utmost importance as these MDR organisms have the potential to spread rapidly in hospital settings and cause nosocomial infections with high death rates (Samra et al., 2007). The diagnosis of carbapenem-resistant organisms has been problem as some of the isolates have a low expression of resistance, which may not be screened via conventional automated and non-automated procedures. *blaKPC* resistance genes have been rapidly detected using molecular techniques on samples taken directly from patients. We have adapted a fast, sensitive, and specific qPCR assay for the detection of *blaKPC* genes in wound samples. The assay can be completed in less than 3 h, which will assist in speeding up the process, thereby decreasing the possibility of the transmission of the organism within hospitals. This is in contrast to the time-consuming, less sensitive, and non-standardized methods of culturing bacteria, which may take over 24 h to identify carbapenem-resistant bacteria (Landman et al., 2005; Solanki et al., 2013). qPCR avoids the possibility of post-PCR contamination and all post-PCR requirements of the method, including gel electrophoresis (Hindiyeh et al., 2005). *blaKPC* genes have been reported in the United States, and further reports have affirmed their presence in Europe, Latin America, China, and India. The capacity of KPC-producing populations to spread through plasmids and other mobile genetic components is of real concern for the horizontal transmission of the gene within Enterobacterales (Chen et al., 2014). These findings concur with those of Albiger et al. (2015), which confirmed the unparalleled predominance of KPC in Asia and the pervasive dispersion of KPC-producing strains that lead the carbapenemase system in the majority of locations.

This study was focused on the *blaKPC* gene rather than on the investigation of other key genes such as *blaNDM*, *blaOXA*, and *blaVIM*, which are also common in India. Multiplex PCR, as well as other multi-gene detection procedures, might therefore provide a more detailed resistance profile. Secondly, this is a single-site study; hence, the results might not represent other areas. Thirdly, no clinical data were gathered that would have demonstrated significant findings pertaining to patient outcomes, length of stay, or treatment response, which would have provided valuable insights into the clinical implications of KPC-producing isolates. Despite such limitations, the critical significance of real-time PCR in daily diagnostic laboratories, especially in high-burden countries, can hardly be ignored. The identification of KPC-positive isolates can prevent the unnecessary use of carbapenem-based empirical therapy by allowing a targeted, prioritized use of empirical therapy and preventing nosocomial outbreaks.

The novelty of this study lies in its demonstration of the utility of a rapid, sensitive, and specific qPCR assay for the detection of the *blaKPC* gene in clinical wound isolates of *K. pneumoniae*. With a turnaround time of less than 3 h and a diagnostic accuracy reflected by an AUC of 0.99, this approach offers a superior alternative to conventional culture-based or phenotypic methods that are often time-consuming and less reliable.

## Conclusion

This study found a significant prevalence of MDR and KPC-producing *K. pneumoniae* in wound infections, with only a few therapeutic choices due to widespread resistance. These findings highlight the importance of the routine implementation of molecular diagnostics such as real-time PCR for the timely identification of high-risk clones. Future research should concentrate on whole-genome sequencing of resistant isolates to identify new resistance determinants, assess clonal propagation, and inform infection control measures.

## Data Availability

The raw data has been deposited here: Zenodo Research data repository Version v110.5281/zenodo.17060580.
