# Supplementary material for: Antimicrobial resistance and real-time PCR detection of blaKPC in Klebsiella pneumoniae isolated from wound infections in a tertiary care hospital
Source: Front Antibiot. 2026 Jan 27;4:1700157. doi: 10.3389/frabi.2025.1700157 (PMC12888029; doi:10.3389/frabi.2025.1700157)
Supplement: Supplementary file 1 [file Table1.docx]

| **S.** **No.** | Clinical Isolates | **MEM** | **CAZ** | **CTX** | **AZT** | **CIP** | **AN** | **AMP** | **TMP** | **TIG** | **TET** | **NIT** | **LEV** |
| --- | --- | --- | --- | --- | --- | --- | --- | --- | --- | --- | --- | --- | --- |
| 1 | KPC001 | 32 | >32 | >32 | >32 | >32 | >32 | >32 | >32 | >8 | >16 | >16 | 32 |
| 2 | KPC002 | >32 | >32 | >32 | >32 | >32 | >32 | >32 | >32 | 2 | 0.5 | 4 | 2 |
| 3 | KPC003 | 4 | 0.25 | 4 | 0.25 | 0.06 | 1 | 8 | 0.25 | 2 | 0.5 | 4 | 2 |
| 4 | KPC004 | >32 | >32 | >32 | >32 | >32 | >32 | >32 | >32 | 4 | 1 | 16 | 16 |
| 5 | KPC005 | >32 | >32 | >32 | >32 | >32 | >32 | >32 | >32 | >8 | >16 | >16 | 16 |
| 6 | KPC006 | >16 | 16 | 16 | 4 | >16 | >16 | >32 | >16 | 2 | 2 | 8 | 4 |
| 7 | KPC007 | >16 | >16 | >16 | >16 | >16 | >16 | >32 | >16 | 2 | 0.125 | 16 | 4 |
| 8 | KPC008 | 8 | >16 | 16 | >16 | >16 | >16 | >32 | >16 | 1 | 0.25 | 16 | 2 |
| 9 | KPC009 | 16 | >16 | >16 | >16 | >16 | >16 | >32 | >16 | 2 | 0.5 | 4 | 0.25 |
| 10 | KPC010 | 4 | 16 | 16 | 0.5 | >16 | >16 | >32 | >16 | 0.5 | 0.125 | 4 | 2 |
| 11 | KPC011 | 8 | 0.125 | 0.03 | >16 | 0.03 | 0.5 | 16 | 0.06 | 0.5 | 0.125 | 8 | 8 |
| 12 | KPC012 | >16 | >16 | >16 | >16 | >16 | >16 | >32 | >16 | 1 | 0.5 | 8 | 16 |
| 13 | KPC013 | 8 | >16 | 16 | 16 | >16 | >16 | >32 | 16 | 1 | 8 | 16 | 32 |
| 14 | KPC014 | 4 | >16 | 4 | >16 | >16 | >16 | >32 | >16 | 2 | 8 | 16 | 32 |
| 15 | KPC015 | 4 | >16 | >16 | 16 | >16 | >16 | >32 | >16 | 0.3 | 4 | 16 | 0.5 |
| 16 | KPC016 | >16 | >16 | >16 | >16 | >16 | >16 | >32 | >16 | >8 | >16 | >16 | 16 |
| 17 | KPC017 | >16 | 16 | 16 | 8 | >16 | >16 | >32 | >16 | 1 | 0.25 | 4 | 0.13 |
| 18 | KPC018 | >16 | >16 | >16 | >16 | >16 | >16 | >32 | >16 | 0.5 | 2 | 8 | 2 |
| 19 | KPC019 | >16 | >16 | >16 | >16 | >16 | >16 | >32 | >16 | 0.5 | 0.125 | 2 | 2 |
| 20 | KPC020 | 8 | 0.06 | 0.03 | 4 | 0.125 | 0.25 | 8 | 0.03 | 1 | 2 | 4 | >16 |
| 21 | KPC021 | 8 | >16 | >16 | 4 | >16 | >16 | >32 | 0.03 | 1 | 8 | >16 | >16 |
| 22 | KPC022 | >16 | >16 | >16 | >16 | >16 | >16 | >32 | >16 | 0.5 | 0.5 | 2 | 0.05 |
| 23 | KPC023 | 4 | 0.06 | 8 | 4 | 1 | 4 | 16 | 1 | 4 | >16 | >16 | 8 |
| 24 | KPC024 | 8 | 8 | 8 | 4 | >16 | 2 | 16 | >16 | 0.3 | 1 | 4 | 8 |
| 25 | KPC025 | >16 | >16 | >16 | >16 | >16 | >16 | >32 | >16 | 2 | 2 | 8 | 16 |
| 26 | KPC026 | 4 | 16 | 16 | 8 | >16 | >16 | >32 | >16 | 2 | 8 | 16 | 8 |
| 27 | KPC027 | >16 | >16 | >16 | >16 | >16 | >16 | >32 | >16 | 8 | >16 | >16 | >32 |
| 28 | KPC028 | 16 | >32 | >32 | >32 | >32 | >32 | >32 | >32 | 0.5 | 2 | 4 | 0.5 |
| 29 | KPC029 | 32 | >32 | >32 | >32 | >32 | >32 | >32 | 1 | 2 | 4 | 16 | 16 |
| 30 | KPC030 | >32 | >32 | >32 | >32 | >32 | >32 | >32 | >32 | 4 | 16 | >16 | >16 |
| 31 | KPC031 | >32 | >32 | >32 | >32 | >32 | >32 | >32 | >32 | 1 | 4 | 16 | >16 |
| 32 | KPC032 | >32 | >32 | >32 | >32 | >32 | >32 | >32 | 1 | 1 | 4 | >16 | >16 |
| 33 | KPC033 | >32 | >32 | >32 | >32 | >32 | >32 | >32 | >32 | 2 | 1 | 4 | 2 |
| 34 | KPC034 | >32 | >32 | >32 | >32 | >32 | >32 | >32 | >32 | 8 | >16 | >16 | 8 |
| 35 | KPC035 | 16 | 16 | >32 | >32 | >32 | >32 | >32 | >32 | >8 | >16 | >16 | 8 |
| 36 | KPC036 | >32 | >32 | >32 | >32 | >32 | >32 | >32 | >32 | >8 | >16 | >16 | >32 |
| 37 | KPC037 | >32 | >32 | >32 | >32 | >32 | >32 | >32 | >32 | >8 | >16 | >16 | 32 |
| 38 | KPC038 | 16 | 16 | 16 | 16 | >32 | 8 | >32 | >32 | 1 | 0.5 | 4 | 2 |
| 39 | KPC039 | 32 | >32 | >32 | >32 | >32 | >32 | >32 | >32 | 2 | 2 | 8 | 8 |
| 40 | KPC040 | 4 | >32 | >32 | >32 | >32 | >32 | >32 | >32 | >8 | >16 | >16 | 8 |
| 41 | KPC041 | >32 | >32 | >32 | >32 | >32 | >32 | >32 | >32 | >8 | >16 | >16 | 8 |
| 42 | KPC042 | >32 | >32 | >32 | >32 | >32 | >32 | >32 | >32 | >8 | >16 | >16 | 8 |
| 43 | KPC043 | 4 | >32 | >32 | >32 | >32 | >32 | >32 | >32 | >8 | >16 | >16 | 8 |
| 44 | KPC044 | >16 | 32 | 32 | 32 | 32 | 32 | >32 | 32 | >8 | >16 | 16 | 16 |
| 45 | KPC045 | >16 | >16 | >32 | >32 | 32 | >32 | >32 | >16 | 2 | >16 | >16 | 32 |
| 46 | KPC046 | >16 | >16 | >32 | >32 | 32 | >32 | >32 | >16 | >4 | >16 | >16 | >32 |
| 47 | KPC047 | >16 | >16 | >32 | >32 | 32 | >16 | >32 | 16 | 2 | >16 | 8 | 16 |
| 48 | KPC048 | 4 | 0.125 | 32 | 0.25 | 0.5 | >16 | 8 | 4 | 2 | 32 | 32 | 16 |
| 49 | KPC049 | >16 | >16 | 32 | >32 | >32 | >16 | >32 | >16 | >4 | 8 | 16 | 16 |
| 50 | KPC050 | >16 | >16 | >32 | 32 | >32 | >16 | >32 | >16 | 4 | 1 | >16 | 16 |
| 51 | KPC051 | 8 | <0.03 | 0.06 | >16 | 0.06 | >16 | 8 | 2 | 4 | 1 | >16 | 8 |
| 52 | KPC052 | >16 | >16 | >32 | >16 | >32 | >32 | >32 | >16 | 1 | >16 | >16 | 8 |
| 53 | KPC053 | >16 | >16 | >32 | >16 | >32 | >32 | >32 | >16 | >4 | >16 | >16 | 16 |
| 54 | KPC054 | >16 | 0.25 | 0.5 | 0.5 | 0.125 | >16 | 4 | 4 | 4 | >16 | >16 | 32 |
| 55 | KPC055 | >16 | >32 | 32 | >16 | >16 | >16 | >64 | >32 | >8 | >32 | >32 | 32 |
| 56 | KPC056 | 4 | >32 | >32 | >16 | >16 | >16 | >64 | >32 | 0.5 | >32 | 16 | 32 |
| 57 | KPC057 | 32 | >32 | >32 | 16 | >16 | >16 | >64 | >16 | >4 | 16 | >32 | 32 |
| 58 | KPC058 | >32 | >32 | >32 | >16 | >16 | >16 | 64 | >16 | >8 | >32 | >32 | >32 |
| 59 | KPC059 | >32 | >32 | >32 | 16 | 0.03 | 0.06 | 8 | >16 | >8 | 16 | >32 | <4 |
| 60 | KPC060 | 16 | >32 | >32 | >16 | >16 | >32 | >64 | >16 | >8 | >32 | 16 | <4 |
| 61 | KPC061 | >32 | >32 | >32 | 8 | 16 | >32 | 64 | >16 | 2 | >32 | >32 | <4 |
| 62 | KPC062 | >32 | >32 | >32 | >16 | >16 | >16 | >64 | >16 | 2 | 16 | >32 | <4 |
| 63 | KPC063 | >32 | >32 | >32 | >16 | >16 | >16 | >64 | >16 | >4 | >32 | >32 | 32 |
| 64 | KPC064 | >32 | >32 | 32 | 4 | 16 | >16 | >64 | >16 | >4 | >32 | >32 | >32 |
| 65 | KPC065 | <2 | 16 | 32 | 4 | 16 | >32 | 8 | 4 | 4 | <2 | >32 | <2 |
| 66 | KPC066 | >32 | >32 | 32 | >16 | >16 | >32 | >64 | >16 | 4 | 4 | <2 | 32 |
| 67 | KPC067 | <2 | >32 | 16 | 4 | >16 | >16 | >64 | >16 | 2 | <2 | >32 | 16 |
| 68 | KPC068 | >32 | >32 | 32 | 4 | 16 | >16 | >64 | >16 | >8 | 8 | >32 | 32 |
| 69 | KPC069 | >32 | >32 | 32 | >16 | >16 | >16 | >64 | >32 | >4 | >32 | >32 | 32 |
| 70 | KPC070 | <2 | <2 | 2 | 8 | 16 | >16 | >64 | >32 | 4 | <2 | >32 | >32 |
| 71 | KPC071 | >64 | >64 | >32 | >16 | 16 | >16 | >64 | >16 | >8 | >64 | >64 | >64 |
| 72 | KPC072 | >32 | >32 | >32 | >32 | >16 | >32 | >64 | >16 | >4 | >32 | >32 | >32 |
| 73 | KPC073 | 16 | >32 | >32 | >32 | >16 | >32 | >64 | >16 | 2 | >32 | >32 | >32 |
| 74 | KPC074 | >32 | >32 | >32 | >32 | >16 | >16 | >64 | >16 | 2 | >32 | >32 | >32 |
| 75 | KPC075 | 8 | >32 | >32 | >32 | >16 | >16 | >64 | >16 | >4 | >32 | >32 | >32 |
| 76 | KPC076 | >32 | 16 | 32 | >32 | >32 | >32 | >64 | >16 | 2 | 4 | >32 | 32 |
| 77 | KPC077 | >32 | 4 | 2 | >32 | >32 | >32 | 32 | >32 | >8 | >16 | >32 | 32 |
| 78 | KPC078 | 4 | >32 | 16 | >32 | >32 | >16 | 32 | >32 | >8 | >32 | >32 | 32 |
| 79 | KPC079 | 8 | >32 | 32 | >32 | >32 | >32 | 32 | >32 | >8 | >32 | >32 | 32 |
| 80 | KPC080 | 8 | >32 | >32 | >32 | >32 | >32 | >64 | >32 | >8 | 32 | >32 | 16 |
| 81 | KPC081 | >64 | >64 | >32 | >32 | >32 | >32 | 64 | >32 | >8 | >64 | >64 | >64 |
| 82 | KPC082 | 8 | <2 | 2 | 16 | >32 | >32 | 16 | >32 | 2 | <2 | >32 | <2 |
| 83 | KPC083 | 4 | >32 | 32 | >32 | >32 | >32 | 32 | >32 | >8 | >32 | 32 | 16 |
| 84 | KPC084 | >32 | >32 | >32 | >32 | >32 | >32 | >64 | >16 | >8 | >32 | >32 | >32 |
| 85 | KPC085 | 16 | >32 | >32 | >32 | >32 | >16 | >64 | >16 | >4 | 32 | 32 | 16 |
| 86 | KPC086 | 8 | >32 | >32 | >32 | >32 | >16 | >64 | >32 | 2 | >32 | >32 | >32 |
| 87 | KPC087 | 16 | 16 | >32 | >32 | >32 | >32 | >64 | >32 | >8 | 16 | >32 | 16 |
| 88 | KPC088 | 8 | >32 | >32 | 32 | >32 | >32 | >64 | >32 | 2 | >32 | >32 | 16 |
| 89 | KPC089 | >32 | >32 | >32 | 32 | >32 | >16 | >64 | >32 | 2 | 2 | >32 | >32 |
| 90 | KPC090 | 16 | >32 | >32 | 32 | >32 | >32 | >64 | >32 | 1 | 2 | >32 | 4 |
| 91 | KPC091 | 32 | >32 | >32 | 16 | >32 | >32 | >64 | >16 | 4 | >32 | >32 | >32 |
| 92 | KPC092 | >32 | >32 | >32 | 32 | 32 | >16 | >64 | >16 | >4 | >32 | >32 | 32 |
| 93 | KPC093 | 32 | >32 | >32 | 32 | 16 | >16 | 32 | >32 | 8 | 2 | >32 | 0.5 |
| 94 | KPC094 | 8 | >32 | >32 | 32 | 32 | >32 | 32 | >32 | >4 | >32 | >32 | >32 |
| 95 | KPC095 | 8 | 0.5 | 0.25 | 32 | 0.125 | >32 | 16 | >32 | 2 | 0.25 | 4 | 0.5 |
| 96 | KPC096 | 32 | 32 | 32 | >32 | 32 | >16 | 32 | >16 | >4 | >16 | >32 | >32 |
| 97 | KPC097 | >16 | >8 | 32 | >32 | 16 | >16 | >64 | >16 | 4 | >8 | >16 | 2 |
| 98 | KPC098 | >16 | >8 | 16 | 32 | 16 | >16 | >64 | >32 | 4 | >8 | >16 | 4 |
| 99 | KPC099 | >16 | >8 | >32 | >32 | 32 | >16 | >64 | >32 | 4 | >8 | >16 | 4 |
| 100 | KPC100 | >16 | >8 | >32 | >32 | >32 | >16 | >64 | >32 | 4 | >8 | >16 | 4 |
| 101 | KPC101 | >16 | >8 | >32 | >32 | >32 | 32 | >64 | >16 | 2 | >8 | >16 | 4 |
| 102 | KPC102 | >16 | >8 | 16 | 32 | >32 | >32 | >64 | >16 | 2 | >8 | >16 | 4 |
| 103 | KPC103 | >16 | >8 | >32 | 32 | >32 | >32 | >64 | >32 | 2 | >8 | >16 | 2 |
| 104 | KPC104 | >16 | >8 | 16 | 32 | >32 | >32 | >64 | >32 | 2 | >8 | >16 | 4 |
| 105 | KPC105 | >16 | >8 | 32 | 16 | >32 | >32 | >64 | >32 | 4 | >8 | >16 | 4 |
| 106 | KPC106 | >16 | >8 | 32 | 16 | >32 | >32 | >64 | >16 | 4 | >8 | >16 | 2 |
| 107 | KPC107 | >32 | >32 | >32 | >32 | >32 | >32 | 64 | >16 | 4 | 0.25 | 16 | >32 |
| 108 | KPC108 | >16 | >32 | >32 | >32 | 0.125 | 0.5 | 8 | 2 | 4 | 0.5 | >32 | 16 |
| 109 | KPC109 | 8 | >32 | >32 | 32 | 16 | 32 | 32 | >16 | >32 | 0.063 | >32 | 16 |
| 110 | KPC110 | >32 | >32 | 16 | >32 | 32 | 32 | 32 | >16 | >32 | 0.5 | >32 | >32 |

Table 1: MIC values of 110 ioslates

| S.No | Clinical Isolate | FAM -bla _KPC_ (Ct value) | CY5 - 16S rRNA (Ct value) |
| --- | --- | --- | --- |
| 1 | KPC001 | 22.5 | 20.3 |
| 2 | KPC002 | 20.3 | 21.3 |
| 3 | KPC003 | 19.8 | 20.6 |
| 4 | KPC004 | 18 | 22.5 |
| 5 | KPC005 | 19.26 | 21.0 |
| 6 | KPC006 | 30.3 | 20.5 |
| 7 | KPC007 | 32.1 | 21.4 |
| 8 | KPC008 | 30.5 | 23.5 |
| 9 | KPC009 | 29.8 | 22.0 |
| 10 | KPC010 | 24.9 | 21.0 |
| 11 | KPC011 | 28.5 | 20.0 |
| 12 | KPC012 | 22.0 | 20.9 |
| 13 | KPC013 | 21.6 | 21.5 |
| 14 | KPC014 | 27.0 | 22.4 |
| 15 | KPC015 | 22.6 | 21.5 |
| 16 | KPC016 | 24.1 | 23.1 |
| 17 | KPC017 | 20.9 | 25.4 |
| 18 | KPC018 | 25.5 | 23.1 |
| 19 | KPC019 | 31.1 | 24.2 |
| 20 | KPC020 | 26.9 | 31.2 |
| 21 | KPC021 | 31.2 | 20.5 |
| 22 | KPC022 | 22.8 | 31.1 |
| 23 | KPC023 | 24.5 | 26.2 |
| 24 | KPC024 | 21.6 | 23.1 |
| 25 | KPC025 | 28.4 | 25.4 |
| 26 | KPC026 | 26.0 | 22.1 |
| 27 | KPC027 | 25.0 | 23.4 |
| 28 | KPC028 | 24.1 | 21.6 |
| 29 | KPC029 | 27.4 | 25.2 |
| 30 | KPC030 | 27.5 | 24.6 |
| 31 | KPC031 | 29.3 | 25.3 |
| 32 | KPC032 | 21.3 | 24.1 |
| 33 | KPC033 | 20.5 | 23.8 |
| 34 | KPC034 | 22.8 | 24.1 |
| 35 | KPC035 | 23.6 | 20.7 |
| 36 | KPC036 | 18.6 | 22.4 |
| 37 | KPC037 | 19.5 | 23.4 |
| 38 | KPC038 | 19.9 | 21.1 |
| 39 | KPC039 | 20.1 | 22.3 |
| 40 | KPC040 | 22 | 24.0 |
| 41 | KPC041 | 24.2 | 25.1 |
| 42 | KPC042 | 19.1 | 25.4 |
| 43 | KPC043 | 31.4 | 22.1 |
| 44 | KPC044 | 29.4 | 21.4 |
| 45 | KPC045 | 26.8 | 21.8 |
| 46 | KPC046 | 30.4 | 21.7 |
| 47 | KPC047 | 27.2 | 20.4 |
| 48 | KPC048 | 25.6 | 20.3 |
| 49 | KPC049 | 19.9 | 20.1 |
| 50 | KPC050 | 29.6 | 22.4 |
| 51 | KPC051 | 30.5 | 23.4 |
| 52 | KPC052 | 33.0 | 25.1 |
| 53 | KPC053 | 30.2 | 24.1 |
| 54 | KPC054 | 28.5 | 22.3 |
| 55 | KPC055 | 21.4 | 21.0 |
| 56 | KPC056 | 20.6 | 20.5 |
| 57 | KPC057 | 22.9 | 26.7 |
| 58 | KPC058 | 24.5 | 21.8 |
| 59 | KPC059 | 29.1 | 25.1 |
| 60 | KPC060 | 27.5 | 20.1 |
| 61 | KPC061 | 30.1 | 22.4 |
| 62 | KPC062 | 32.1 | 24.6 |
| 63 | KPC063 | 28.5 | 26.1 |
| 64 | KPC064 | 20.5 | 25.2 |
| 65 | KPC065 | 23.4 | 22.9 |
| 66 | KPC066 | 22.7 | 20.5 |
| 67 | KPC067 | 25.8 | 21.5 |
| 68 | KPC068 | 26.4 | 30.6 |
| 69 | KPC069 | 22.5 | 32.1 |
| 70 | KPC070 | 23.1 | 31.0 |
| 71 | KPC071 | 20.8 | 25.6 |
| 72 | KPC072 | 30.1 | 22.1 |
| 73 | KPC073 | 19.6 | 22.8 |
| 74 | KPC074 | 20.5 | 23.8 |
| 75 | KPC075 | 22.4 | 24.1 |
| 76 | KPC076 | 23.5 | 20.9 |
| 77 | KPC077 | 28.1 | 26.4 |
| 78 | KPC078 | 24.0 | 25.2 |
| 79 | KPC079 | 26.5 | 23.7 |
| 80 | KPC080 | 25.4 | 22.8 |
| 81 | KPC081 | 21.9 | 21.9 |
| 82 | KPC082 | 22.3 | 20.4 |
| 83 | KPC083 | 28.1 | 21.9 |
| 84 | KPC084 | 26.4 | 26.5 |
| 85 | KPC085 | 30.9 | 25.4 |
| 86 | KPC086 | 29.4 | 24.3 |
| 87 | KPC087 | 21.0 | 22.0 |
| 88 | KPC088 | 20.6 | 22.9 |
| 89 | KPC089 | 22.0 | 23.5 |
| 90 | KPC090 | 28.1 | 24.1 |
| 91 | KPC091 | 32.5 | 23.6 |
| 92 | KPC092 | 25.0 | 25.4 |
| 93 | KPC093 | 31.4 | 21.4 |
| 94 | KPC094 | 28.1 | 24.7 |
| 95 | KPC095 | 23.5 | 22.6 |
| 96 | KPC096 | 24.6 | 21.3 |
| 97 | KPC097 | 29.1 | 25.1 |
| 98 | KPC098 | 29.1 | 23.4 |
| 99 | KPC099 | 20.17 | 31.2 |
| 100 | KPC100 | 26.5 | 30.5 |
| 101 | KPC101 | 22.3 | 27.1 |
| 102 | KPC102 | 28.4 | 21.4 |
| 103 | KPC103 | 26.1 | 32.5 |
| 104 | KPC104 | 31.2 | 29.5 |
| 105 | KPC105 | 22.6 | 27.1 |
| 106 | KPC106 | 25.1 | 28.1 |
| 107 | KPC107 | 22.7 | 29.5 |
| 108 | KPC108 | 20.1 | 20.5 |
| 109 | KPC109 | 19.5 | 22.6 |
| 110 | KPC110 | 18.9 | 24.5 |
| Positive Control | | 22.1 | 24.6 |
| No Template control | | Not detected | Not detected |

Table 2: **Ct Values of FAM (bla _Kpc_  ) And Cy 5 ( 16s RNA) of 110 ioslates**


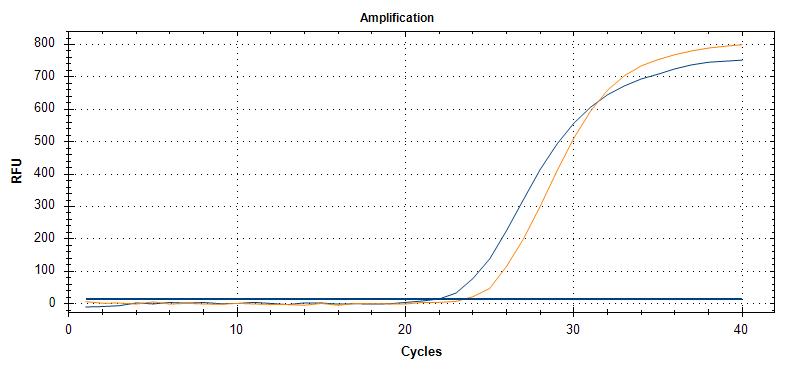


**Figure 1. Positive control KPC -FAM 16Sr RNA Cy5**


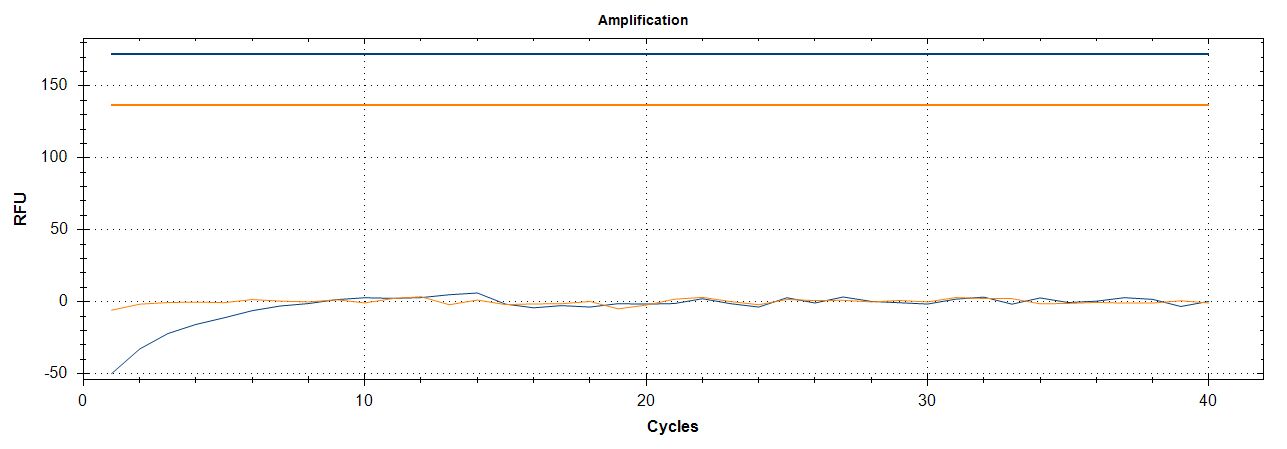
 Figure 2 **Negative control KPC -FAM 16Sr RNA Cy5**


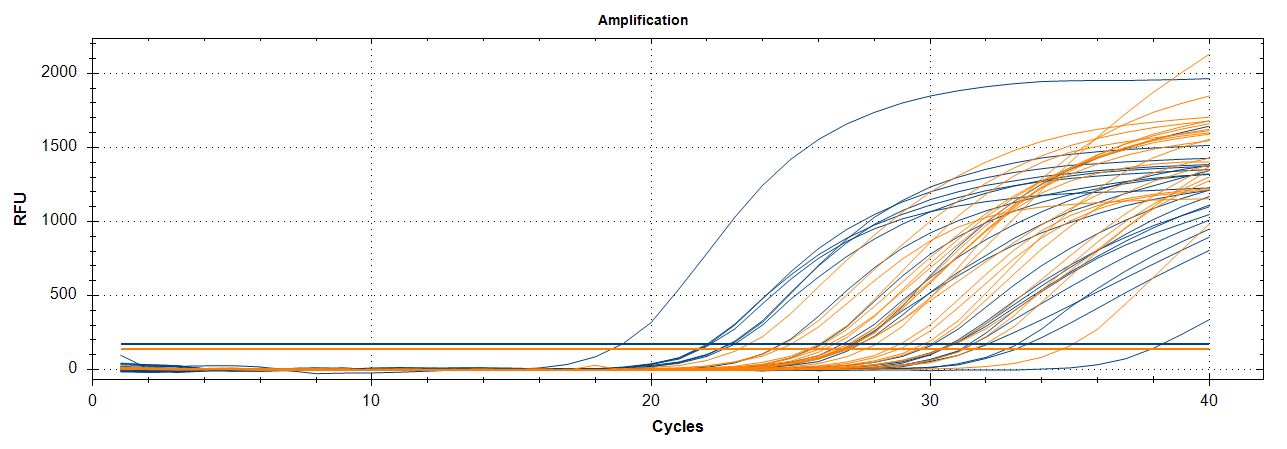
**Figure 3: KPC and 16srRNA amplification of samples**
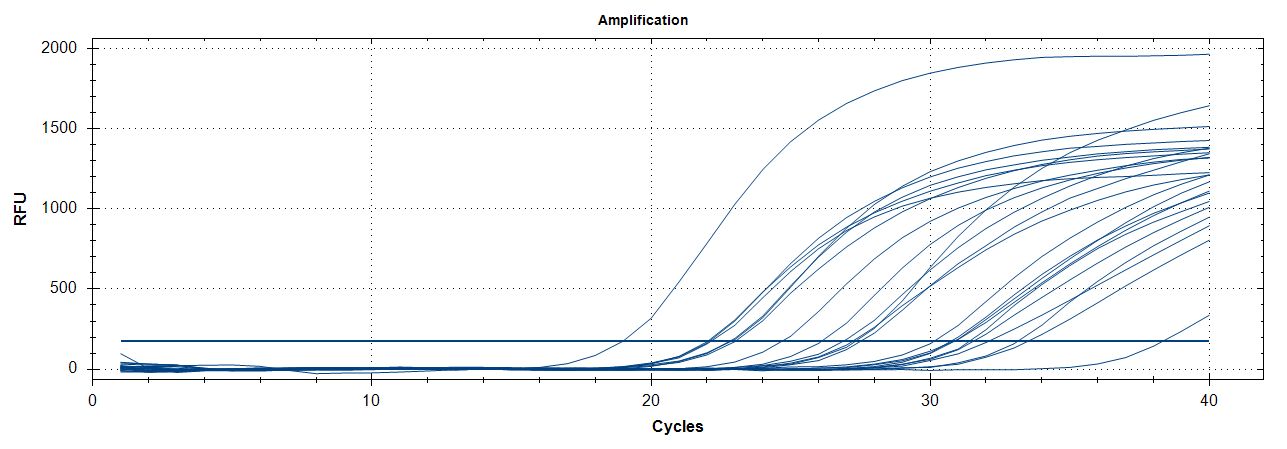
Figure 4 : **KPC gene amplification of the isolates**


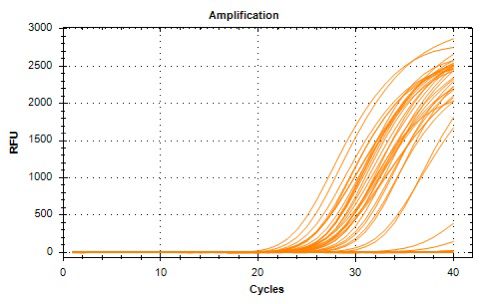


Figure 5: **16srRNA amplification of the ioslates**
